# Extremely stretchable and conductive water-repellent coatings for low-cost ultra-flexible electronics

**DOI:** 10.1038/ncomms9874

**Published:** 2015-11-23

**Authors:** Joseph E. Mates, Ilker S. Bayer, John M. Palumbo, Patrick J. Carroll, Constantine M. Megaridis

**Affiliations:** 1 Mechanical and Industrial Engineering, University of Illinois at Chicago, Chicago, Illinois 60607, USA; 2 Smart Materials, Nanophysics, Istituto Italiano di Tecnologia, 16163 Genoa, Italy

## Abstract

Rapid advances in modern electronics place ever-accelerating demands on innovation towards more robust and versatile functional components. In the flexible electronics domain, novel material solutions often involve creative uses of common materials to reduce cost, while maintaining uncompromised performance. Here we combine a commercially available paraffin wax–polyolefin thermoplastic blend (elastomer matrix binder) with bulk-produced carbon nanofibres (charge percolation network for electron transport, and for imparting nanoscale roughness) to fabricate adherent thin-film composite electrodes. The simple wet-based process produces composite films capable of sustained ultra-high strain (500%) with resilient electrical performance (resistances of the order of 10^1^–10^2^ Ω sq^−1^). The composites are also designed to be superhydrophobic for long-term corrosion protection, even maintaining extreme liquid repellency at severe strain. Comprised of inexpensive common materials applied in a single step, the present scalable approach eliminates manufacturing obstacles for commercially viable wearable electronics, flexible power storage devices and corrosion-resistant circuits.

## Abstract (web summary)

The development of modern flexible electronics calls for new materials with extreme stretchability and high conductivity. Here, Mates *et al*. show a printable material made of commercially-available elastomers and carbon nanofibres, which exhibits low resistance and water-repellency at strain up to 500%.

## Graphical abstract

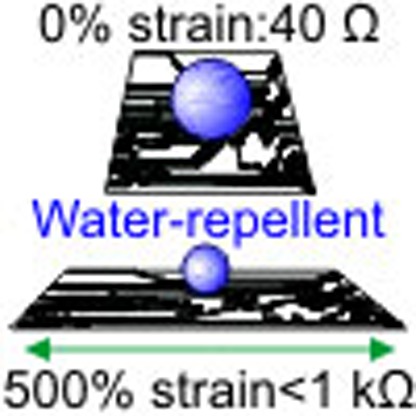

The increasing complexity and seamless integration of modern electronics require innovative material solutions to address rising demands for greater flexibility and improved functionality. Extreme stretchability and low electrical resistance are often mutually exclusive in flexible electronics applications. Only recently, elastomeric conductive composites have shown promise for sustainable high-strain and recoverable conductivity^1^,^2^,^3^,^4^,^5^,^6^, with increased interest specifically in flexible battery applications^7^,^8^,^9^. Separately, water-repellent (that is, superhydrophobic) conductive composites have been investigated for operation under wet or corrosive conditions^10^,^11^,^12^ with potential for extending component lifetime. Combining stretchable and superhydrophobic functionalities in an electrode material is advantageous for many electronics applications, such as flexible energy storage, wearable electronics and printable circuitry. While examples^13^,^14^ of such multifunctional conductive materials exist in the literature, their elastic recovery has rarely been demonstrated for stretch ratios beyond *λ*>3 (*λ*=final length/initial length, or *L*_f_/*L*_i_). More specifically, a stretchable, conductive and water-repellent material that maintains functionality at *λ*=6 (that is, 500% strain) has not been achieved to date^15^,^16^,^17^,^18^,^19^.

The large-area fabrication of these thin composites is achieved via simple methods incorporating Parafilm-M (PF) and electrically conductive carbon nanofibres (CNFs). The rapid-drying composites are sprayed from a colloidal dispersion to deliver low-cost, substrate-independent thin films suitable for electrode and circuitry components operating in wet environments (where metals are most susceptible). Performance exceeds previous benchmarks^6^,^9^,^20^,^21^ for flexible elastomeric composites of either conductive or superhydrophobic property, yet simultaneously attaining both properties. Moreover, these promising and demonstrably resilient composites show recoverable performance under cyclical strain and are attractive for the manufacture of commercially viable electronic products such as wearable electronics, flexible power storage devices and corrosion-resistant circuits.

CNFs have been used extensively in polymer composites since their re-discovery in 1991 (ref. 22), having been shown effective for high conductivity^23^,^24^,^25^, enhanced heat transfer^26^,^27^,^28^, electromagnetic shielding^29^,^30^ and liquid repellency^31^,^32^ applications, among others. CNFs were incorporated in the present composites for their unique ability to form excellent charge percolation networks, even under mechanical strain, by virtue of their high aspect and surface area-to-volume ratios (∼10^8^)^33^,^34^,^35^. Similar to carbon nanotubes in morphology, yet generally an order of magnitude less expensive, CNFs offer re-entrant surface texture ideal for robust superhydrophobic surfaces^36^. Other carbonaceous materials have been used to dope paraffin wax for enhanced heat transfer applications^37^,^38^, but an in-depth characterization of paraffinic conductive composites for their elastomeric potential is lacking in the literature.

PF is a paraffin wax–polyolefin thermoplastic blend used in nearly all modern laboratories as a stretchable vapour barrier to prevent solvent evaporation and/or contamination. The versatile stretchable and adhesive properties of PF are well-studied, and as such, PF has found application in grafting scaffolds for plants^39^,^40^, gas chromatography^41^, as well as medical testing^42^,^43^,^44^. To the best of our knowledge, there have been no published reports of either solution-processing PF for use in nanomaterial composite systems, or embedding conductive fillers in PF to generate flexible electronic systems. The blend of paraffin waxes and polyolefins comprising PF produces a hydrophobic and self-adherent substrate, which displays irreversible elongational thinning as the material is stretched. The good adhesion of the PF polymer matrix to natural rubber enables the irreversible elongation of the PF composite to become reversible. Upon relaxation from a high-strain state, the adhesive and cohesive properties of the composite maintain the charge percolation network of CNFs and their hierarchical micro/nanoscale surface roughness. Of note, such robust and recoverable water repellency is achieved without the use of fluorinated chemistry, an increasingly common industrial requirement in the pursuit of environmentally benign ‘green' products due to an adverse accumulation and bio-persistence of fluorinated compounds in humans and other living organisms (for example, perfluorooctanoic acids)^45^,^46^.

Functional composites from spray have proven viable for scalable substrate-independent application^47^,^48^,^49^,^50^,^51^. Multifunctional CNF–PF composites were spray coated onto natural rubber strips and characterized for elastomeric, superhydrophobic and electroconductive properties. The choice of natural rubber substrates enabled testing of material properties before and after extreme elastic deformation. The substrates were observed to mechanically fail above *λ*=7 (seven times their original size), thus a maximum *λ*-value of 6 was established as the upper bound for much of our testing.

The multifunctional performance of these composites is demonstrably high for both electroconductive and water-repellent applications. Such deformable electrode films can be utilized in a wide variety of non-wettable, corrosion-resistant electronic devices under high strains, forming a versatile platform for the design of future electrical systems with tunable functionality (for example, cathode, anode). We present structurally resilient and electrically conductive composite coatings capable of sustained elongations up to *λ*=6, delivering low sheet resistance and super repellency to water under maximum strain. These thin multifunctional composite electrodes are not only novel for their superior performance, but also comprise inexpensive common materials applied in one step with an ultra-facile and scalable wet-based approach.

## Results

### Scanning electron microscopy

**Figure 1:**
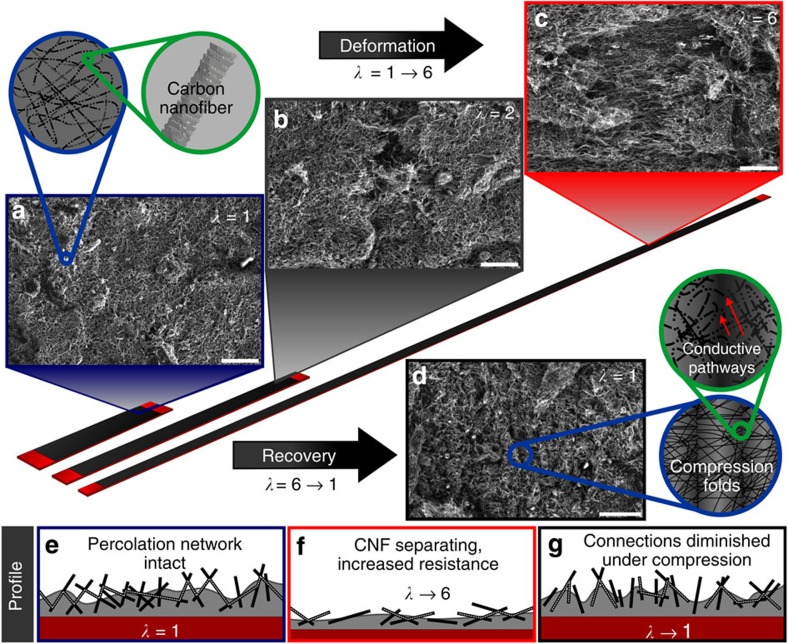
Schematic representation of stretch mechanism with accompanying SEM images. Stretch sequence of a 50 wt.% CNF (*ϕ*=0.5) composite coating applied on a natural rubber substrate (scale bars, 25 μm): (**a**) before the stretch cycle at *λ*=1, (**b**) *λ*=2, (**c**) *λ*=6 and (**d**) after relaxing to an unstretched state, *λ*=1 (schematic illustrations of coated rubber substrates (dark red) are drawn to relative scale). As the stretched sample recovers from deformation, folds in the composite form as a result of its strong adhesion to the substrate. The charge percolation network of CNFs is reinvigorated across these folds, resulting in recoverable electrical performance. (**e**,**f**) Profile view of stretching routine; CNFs (black lines) embedded in PF (grey matrix) on the rubber substrate (dark red). (**e**) Initial profile view of an uninterrupted electrical path (white dashed line). (**f**) CNFs separate during stretching, thus raising electrical resistance. (**g**) Incomplete conductive network recovery after stretch relaxation (compression).

Figure 1 illustrates a 50 wt.% CNF (particle filler mass divided by total solids mass, or *ϕ*=0.5) composite surface on rubber through a full stretch cycle (*λ*→6→1) as observed via scanning electron microscopy (SEM): before stretching (*λ*=1, Fig. 1a), at two points of sustained strain (*λ*=2 and 6, Fig. 1b,c, respectively), and after the composite was returned to an unstretched state to assess morphological recovery of the surface (*λ*=1, Fig. 1d). Figure 1 also contains schematic illustrations of the coated rubber substrates drawn to relative scale accompanying the SEM images. In Fig. 1a, the high level of re-entrant surface roughness from the overlapping tangled cylindrical CNFs (inset) is evident in the composite along with hierarchical microstructures formed by larger CNF–PF clusters. Roughness generated from randomly oriented CNFs also results in a highly effective charge percolation network. Thus, any current travelling through the composite experiences low resistance due to the large number of charge-conducting pathways; nominally seeking the path of least electrical resistance.

In the stretched states shown in Fig. 1b,c, the direction of stretch becomes evident as lateral fissures formed and greater CNF alignment appeared in the composite; particularly evident in the extreme *λ*=6 state. The fully relaxed state (*λ*=1) in Fig. 1d indicates the charge percolation network has recovered to an extent as the fissures observed in Fig. 1c have closed, allowing CNF re-connections to occur across compression folds in the composite. The inset of Fig. 1d shows a schematic of the composite surface after returning to a relaxed state (*λ*=1) resembling an accordion-like collapse. These collapsed folds are primarily a result of irreversible PF elongation, overcome in part by the excellent cohesion and adhesion of the composite to itself and the rubber substrate. The reversible elastomeric property of the underlying natural rubber substrates combined with the polymer's adhesion induces composite compression upon relaxation from stretch. Figure 1d shows the sample-retained nanoscale morphology remarkably similar to its original non-stretched composite surface (Fig. 1a), yet with evident fold lines (oriented vertically in Fig. 1d) formed perpendicular to the direction of stretch (horizontal in this image). Although the charge percolation network became more tortuous as a result of the extreme stretch, repaired electronic pathways (re-connected CNF conduits) across folds in the relaxed composite (Fig. 1d, inset) impart conductive self-healing properties. Figure 1e–g depicts schematically the CNF–PF percolation network during sample stretch/recovery, discussed later in conjunction with electrical resistance results.

### Electrical performance

**Figure 2:**
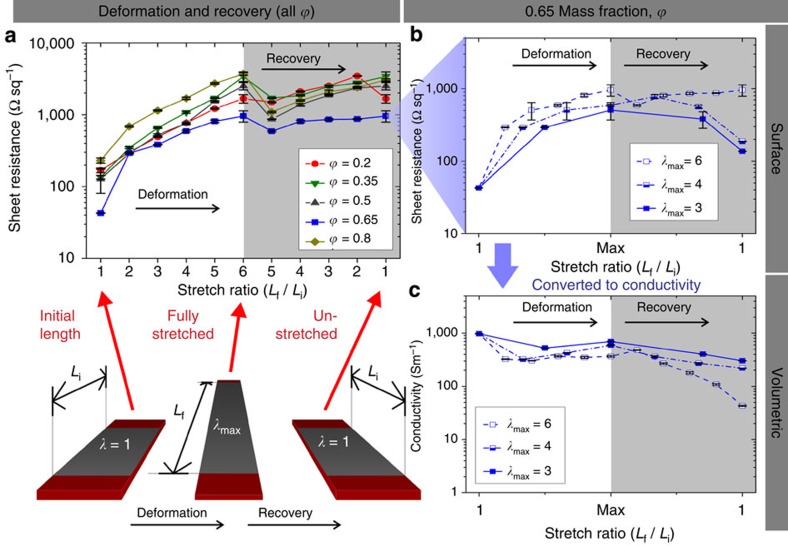
Electrical performance through one full stretch cycle with schematic illustration. (**a**) Sheet resistance values for all *ϕ*, through one full stretching cycle from *λ*=1 to 6, then returned to a relaxed state (*λ*=6→1). The *ϕ*=0.65 composite shows the best performance recovery after the stretch cycle, and was thus chosen for further testing at lower *λ*_max_. (**b**) The *ϕ*=0.65 composite was tested at lower maximum stretch ratios of *λ*_max_=4 and 3 to compare with the extreme *λ*_max_=6 case. Stretched composites with *λ*_max_ of 4 and 3 revealed a much better recovery performance, suggesting *λ*_max_=4 to be the upper limit of sustainable strain with fully recoverable properties (error bars represent s.d. from average resistance values). (**c**) The sheet resistance values for the *ϕ*=0.65 composite were converted to volumetric conductivity using profile SEM images (Supplementary Fig. 1) to obtain coating thickness (Supplementary Fig. 2). The schematic in lower left is an illustration of coated rubber substrates as they transition from initial length (*λ*=1), to maximum deformation *λ*_max_, and back to *λ*=1.

CNF mass fraction (*ϕ*) in the composites was varied from low to high (*ϕ*=0, 0.2, 0.35, 0.5, 0.65 and 0.8). Electrical resistance for each *ϕ* was measured on multiple samples as a function of instantaneous stretch ratio, *λ*, from the original unstretched state (*λ*=1), up to a maximum stretch at *λ*=6, and after returning to *λ*=1 through relaxation, as shown in Fig. 2 (*ϕ*=0 composites were naturally non-conductive). Figure 2a shows that at the initial (unstretched) state *λ*=1, sheet resistance declines monotonically with CNF content rising from *ϕ=*0.2 to 0.65; diminished resistance is attributed to the rising number of percolative CNF contacts at higher *ϕ* (with the exception of *ϕ*=0.8). As samples were stretched, resistance increased with deformation and peaked at *λ*=6. Subsequently, as each composite relaxed back down from *λ*=6 to 5, resistance dropped for all composite ratios, suggesting a re-invigoration of the conductive CNF network previously oriented along fissures (Fig. 1c) in the stretch axis direction (where conductive pathways were diminished). During this interval of relaxation between *λ*=5 to 1, resistance rose (with the exception of only the *ϕ*=0.2 composite, which contained the lowest amount of conductive filler) until performance closely matched the highest observed resistance at maximum stretch. This is indicative of irreversible damage occurring at high strain, suggesting a lower allowable *λ*_max_ to achieve better electrical performance and recovery. Nonetheless, even the highest sheet resistance values seen in Fig. 2a do not exceed 4 kΩ sq^−1^, while remarkably, the highest performing composite (*ϕ*=0.65) achieves sheet resistance below 1 kΩ sq^−1^ at *λ*=6.

Elaborating on sample resistance variability during stretch and recovery (or self-healing) of the charge percolation network, we revert to Fig. 1e–g. In the initial unstretched state (Fig. 1e, *λ*=1), the percolation network is intact as formed during spray deposition. In the intact network, there exist multiple avenues of charge transport via inter-connected CNF pathways through the PF matrix. During the composite stretch to *λ*=6 (Fig. 1f), CNFs realign along the laterally extending substrate as the matrix thins. CNF spacing also increases during elongation, damaging/reducing CNF connections and increasing electrical resistance. In Fig. 1g, after relaxation of the composite back to an unstretched state (*λ*→1), some charge transport pathways are restored (that is, self-healing through strong adhesion to elastomeric substrate). Compression folds from the irreversible thinning of the PF matrix collapse against one another as they are forced together by substrate contraction. As folds develop, CNFs extending away from the surface re-orient as they adjust to fold morphology. CNF re-orientation restores some percolative contacts to reduce resistance from that at *λ*_max_, but there is an overall slight increase from initial film resistance (Fig. 2) depending on the severity of *λ*_max_.

Regarding the unique performance of the ‘book-end' *ϕ*=0.2 and *ϕ*=0.8 composites, the role of filler content must be considered; not only in terms of charge transport, but also in relation to composite formation and stability. For enhanced charge transport, the more evenly balanced composites (*ϕ*=0.35, 0.5 and 0.65) perform as expected with a natural reduction in sheet resistance as a function of increased filler content, with *ϕ*=0.65 representing optimal electronic properties. Yet, a deviation from intuitive electronic performance is observed for both the binder-dominated (*ϕ*=0.2) and filler-dominated (*ϕ*=0.8) composites.

As expected from its low conductive filler content, initial sheet resistance for *ϕ*=0.2 is higher than that observed in the *ϕ*=0.35, 0.5 and 0.65 composites. However, under stretch (*λ*>1) and after full relaxation (*λ*→1), the *ϕ*=0.2 sample outperforms all but the *ϕ*=0.65 composite in terms of low resistance. This anomalous behaviour is believed to be due to the high polymer content delivering a smoother composite surface. CNFs in higher *ϕ* samples actually extend out of the composite surface to form charge pathways away from the bulk, giving the surface a ‘furry' appearance (Supplementary Fig. 1). During spray deposition, much of the CNFs and their corresponding charge-conducting contacts are on the interior of the *ϕ*=0.2 composite, resulting in a non-‘furry' surface. This unique morphology for *ϕ*=0.2 is mentioned again with regards to its impact on wettability in the results for liquid repellency. As the composite is stretched, the realignment of CNFs parallel to the direction of stretch is more pronounced than in higher mass fractions, thus resulting in reduced losses of percolative contact points as the filler majority attains only one direction of alignment versus two. In other words, a lack of orientation perpendicular to the substrate plane results in more electrical contacts within the composite itself, parallel to the substrate.

Conversely, the resistance of the *ϕ*=0.8 composite is highest comparatively; this is attributed to the minimal presence of PF which acts as a binder for CNFs to maintain percolative contact. In addition, the abundance of CNFs in the *ϕ*=0.8 case results in agglomerative fibre clumping during the dispersion step, thus reducing spatial uniformity of percolative contacts upon solvent evaporation and solid film formation. In turn, higher resistance is observed compared with other *ϕ*-values. In fact, high-aspect-ratio nanostructured carbon materials such as carbon nanotubes and CNFs can uniformly disperse in thermoplastic matrices up to 30 wt.%, as demonstrated by Koerner *et al*.^52^. Reduced polymer content in *ϕ*=0.8 thus results in poor adhesion of filler within the composite and to the substrate itself. Resultant films are ‘dusty' and non-uniform, as there is insufficient polymeric binder for efficient filler dispersion or filler adhesion, thus delivering the worst electrical performance despite having the highest *ϕ*-value tested.

In real-world applications of flexible electronic components, elastic deformations of *λ*=6 are well beyond most robustness expectations^18^. Lipomi *et al*.^53^ produced conductive, stretchable nanotube films by spray-coating single-walled nanotubes onto poly(dimethylsiloxane) from a solution in *N*-methylpyrrolidone. They also spin coated a solution of charge transfer dopant (tetrafluorotetracyanoquinodimethane in chloroform) over the films to obtain conductivities as high as 2,200 S cm^−1^ at *λ*=2.5. These conductors were not tested above this limit. The pioneering work of Lee *et al*.^15^ presented stretchable metal electrodes featuring ultra-long metal (Ag) nanowire networks that showed recoverable sheet resistance (9–70 Ω sq^−1^, unstretched) after being stretched over *λ*=5.6 (50–400 Ω sq^−1^, fully stretched). However, fabrication of these relatively expensive electrodes required multiple steps, including filtration, film transfer and thermal annealing at 220 °C; in addition to electrode pre-straining (to *λ*=4) to withstand the excessive strains. Woo *et al*.^17^ also used ultra-long Ag nanowire/single-walled carbon nanotube hybrid conductors embedded in a polymer (Ecoflex) matrix by plasmonic welding and formed into stretchable electrodes that withstood stretching to *λ*=5.8 without pre-straining. However, conductivity was not recoverable after stretching to *λ*=5.8. Zang *et al*.^16^ reported large-area conductive coatings made of graphene that were bi-axially crumbled to create folds facilitating moderate resistance (few kΩ) at *λ*=5.5. Although the graphene coating was also superhydrophobic in the pre-strained state, water repellency was gradually lost with stretching. Remarkably, electrical resistance rose by less than a factor of 2 when stretched at *λ*=5.5.

The high stretch ratio of *λ*=6 was chosen here to gauge composite resilience and enable determination of an ideal mass fraction for further testing. From Fig. 2, the 0.65 mass fraction composite offers the optimal CNF to PF ratio for electrical resistance under strain. Composites with lower CNF content possess less efficient charge percolation, while composites with higher CNF content do not contain enough pliable adherent binder (PF) and are thus more vulnerable at high strain. The mass fraction *ϕ*=0.65 was thus selected for further characterization at two lower max stretch ratios, *λ*_max_=4 and 3. The samples demonstrate greater performance recovery when *λ*_max_≤4, as shown in Fig. 2b,c. Sheet resistances of stretched composites at maximum *λ*-values of 3 and 4 recover to 1.8 × and 2.6 × their original sheet resistance, respectively. See Supplementary Fig. 3 for a plot of sheet resistance changes relative to pre-stretch resistance (Δ*R*/*R*_o_) versus stretch ratio for *ϕ*=0.65.

### Thermal characterization

Heating concerns are especially valid when discussing applications such as flexible energy storage where run-away heating events can lead to physical damage for a device. As such, understanding the upper bounds of tolerable temperatures before degradation or melting of the present composites is necessary. When considering the effect of Joule heating on the polymeric matrix under projected power usage in the presence of high strain, differential scanning calorimetry (DSC) enabled a determination of transition temperatures for the polymer matrix, as shown in Supplementary Fig. 4. The wax component of PF was extracted with chloroform and the DSC heating curve of the extracted wax (not shown here) indicated two endothermic peaks at 74 and 95 °C. These peaks are the result of melting crystalline wax fractions having different molecular weight distributions^54^. As these peaks strongly overlapped, it was not possible to calculate melting enthalpies of the different fractions. In PF, however, only one endothermic peak at 80 °C was observed, in addition to the polyolefin melting peak (106.7 °C). The position of this peak did not correspond to any of the extracted wax peaks (74 and 95 °C), but fell between those observed for the melting–solidification transitions of the extracted wax. The DSC heating curve of PF indicates co-crystallization of wax and polyolefin. In other words, it is highly probable that a large part of the wax chains in PF co-crystallized with polyolefin, leaving some of the wax chains to crystallize separately forming a crystalline fraction different from those originally present in pure wax. This indicates some level of miscibility in the melt, which is considered advantageous against potential wax leaching under Joule heating. If the wax component of PF were not miscible with the polyolefin compound, it would have leached (remained at a liquid state) under certain Joule heating conditions limiting the usability of this stretchable conductor. It must be noted that the presence of the CNFs in the PF matrix imposes certain changes on the crystallinity of the matrix as demonstrated by Koerner *et al*.^52^; however, in-depth analysis of such effects is beyond the scope of the present work.

Using conservative min/max resistance values for the *ϕ*=0.65 composite of either 40 Ω or 1 kΩ (*λ*=1 or 6, respectively), power can be determined for a steady 2 A current to be 160 W (*λ*=1), or 4 kW (*λ*=6). Conservatively assuming no heat dissipation and constant use at this power, the electrode can be in operation for ∼16 min in its unstretched state, or only 38 s at *λ*=6 before it reaches 80 °C (transition temperature of the composite). Of course, heat dissipation is inevitable and remains a major design concern for all practical electronics. It is also highly unlikely the device would be required to operate at maximum stretch (that is, worst-case scenario) for long periods. Thus, the present composite coatings could operate safely at currents up to 2 A without physical damage at low *λ*-values for an indefinite period, and high *λ*-values for short intervals, assuming dissipative heat transfer is maintained at or above 160 W m^−2^.

### Liquid repellency

**Figure 3:**
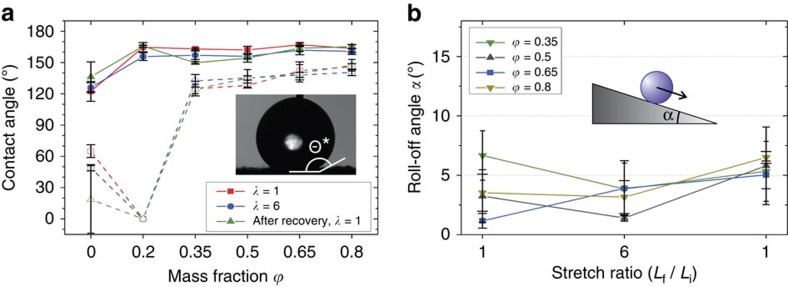
Characterization of liquid repellency. (**a**) Advancing (solid lines, *θ*_Advancing_) and receding (dashed lines, *θ*_Receding_) water contact angles as a function of *ϕ*, through a full stretch cycle (*λ*=1→6→1); superhydrophobic performance remains relatively unchanged through extreme stretch conditions (error bars represent s.d. from average CA measurements). Contact angle hysteresis, defined as *Δθ*=*θ*_Advancing_– *θ*_Receding_, remains low for *ϕ*≥0.35, which is indicative of robust liquid repellency. (**b**) Water droplet roll-off angle data for the same stretch cycle (error bars represent s.d. from average roll-off angle measurements). With the exception of the *ϕ*=0.2 composite (no roll-off), all α-values were <10°, demonstrating high droplet mobility on the surfaces, even under maximum strain. The *ϕ*=0.2 composite was ‘sticky,' attributed to a high CA hysteresis (low receding CA reflects high droplet adhesion to the surface).

A durable and extremely elastomeric composite material having high conductivity and water repellency has potential in adaptable, corrosion-resistant electronics, or any application where a flexible electrode material is required. Water repellency under dynamic stretch conditions for the present composites is displayed and quantified in Fig. 3. Advancing/receding contact angle (CA) (Fig. 3a) and droplet roll-off angles, *α* (Fig. 3b), were measured as a function of CNF mass fraction for the unstretched (*λ*=1), maximum stretch (*λ*=6) and fully relaxed (*λ*=6→1) states. The *ϕ*=0 composite (0 wt.% CNFs) represents an all-PF coating on natural rubber, providing the control case for evaluating enhancement to water repellency imparted by increased CNF content. For all composites (*ϕ*>0), advancing CA remained above the commonly accepted threshold for superhydrophobicity (150°). Receding CA for all but the 0.2 mass fraction composite approached ∼140° as mass fraction increased, thus delivering low CA hysteresis (Δ*θ*=*θ*_Advancing_–*θ*_Receding_<20°) indicative of extremely low-water droplet adhesion to the composite surface (attributed to the nanoscale roughness of CNFs combined with the low surface energy of PF). This is verified by the corresponding low droplet roll-off angles, *α*<7° (Fig. 3b).

Dynamic CA and roll-off angle measurements indicate composite repellency is negligibly affected by extreme strain. Even through maximum elastic deformation (*λ*=6), robust superhydrophobic performance is retained. Retention of the superhydrophobic property after such severe mechanical deformation has not been previously demonstrated, and offers a technological advancement in flexible repellent composites. Uniquely, the *ϕ*=0.2 coating delivered 0° receding CA with no observable roll-off angle. This anomalous behaviour, similar to the non-intuitive electrical performance, is likely due to low CNF concentration resulting in inadequate surface roughness. Limited roughness can provide surface asperities for liquid droplets to anchor and adhere (that is, pinning) as compared with the relatively smooth all-PF surface (*ϕ*=0). At higher *ϕ*, roughness contributes to an area reduction in the liquid–solid interface, allowing droplets to sustain a Cassie–Baxter state^55^, thereby resting on pockets of entrained air and able to glide across the surface under low tilt, or roll-off angles (*α* in Fig. 3, right).

### Durability testing

**Figure 4:**
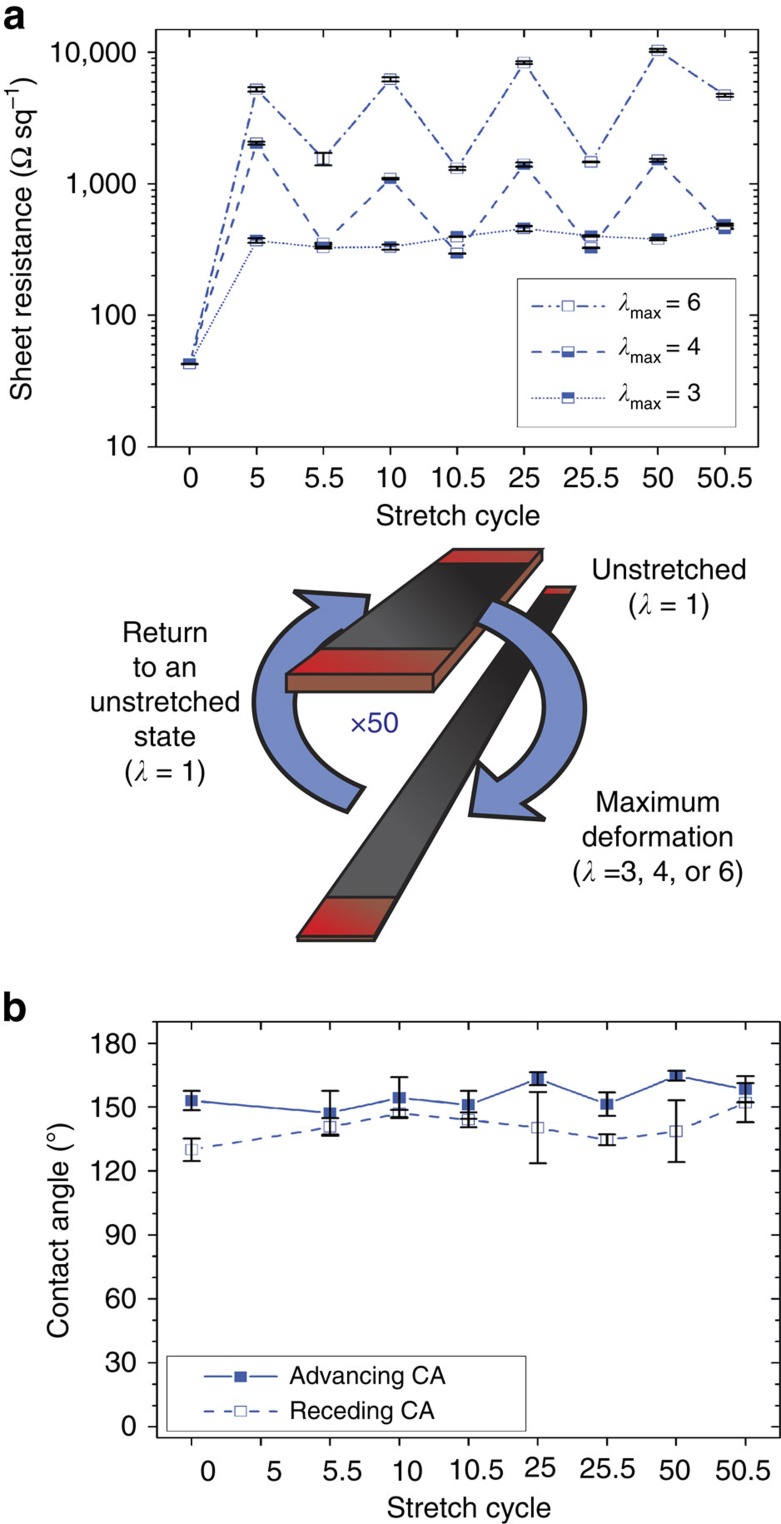
Durability testing for electrical performance and liquid repellency. The best-performing *ϕ*=0.65 composite was tested under 50 deformation cycles to determine resilience after prolonged use and excessive deformation. (**a**) Electrical sheet resistance (error bars represent s.d. of 4-probe resistance measurements), and (**b**) advancing/receding CA measurements were determined at stretch intervals 5, 10, 25 and 50 throughout 50 full stretch cycles for *λ*_max_=6, 4 and 3 (error bars represent s.d. for CA measurements). Half cycle intervals (5.5, 10.5 and so on) represent the composite returned to a relaxed state (*λ*=1) before the commencement of the next deformation cycle.

Figure 4 demonstrates the durability of the best-performing *ϕ*=0.65 composite through multiple strain cycles. The composite was cycled through 50 stretch routines from *λ*=1 to 6, and back to *λ*=1 for each full cycle. Electrical sheet resistance (Fig. 4a) and advancing/receding CA (Fig. 4b) were measured as a function of stretch cycle to determine resilience of the composite under excessive and repeated deformation. Performance was characterized at discrete intervals, namely at 5, 10, 25 and 50 stretch cycles. The end of each full stretch cycle is denoted as a half interval on the *x* axes of the plots (that is, 5.5, 10.5 and so on), where the composite was returned to an unstretched state (*λ*=1) before beginning another stretch cycle. The *R*_s_ values for the *λ*_max_=6 cycles show gradual (albeit not precipitous) degradation in performance through deformation cycling; as such, the max stretch ratio was reduced to ascertain performance recovery at lower *λ*_max_ values. Recovery performance improved considerably when *λ*_max_ was reduced to 4 over 50 cycles, and even more so for *λ*_max_=3 (Fig. 4a). The variation in *R*_s_ for *λ*_max_=3 through 50 cycles is minimal with the primary damage to electrical properties occurring after the first stretch, and *R*_s_ remaining stable for every cycle thereafter. The modulation of the *λ*_max_=4 stretch performance suggests that a change in the percolation network is encountered, passing a critical probability threshold for charge-conducting pathways. The slight improvement in performance of the *λ*_max_=3 over *λ*_max_=4 at intervals is likely due to the realignment of CNFs, as mentioned in Fig. 1e–g and the accompanying discussion, further strengthening the hypothesis of self-healing electrical properties. The superior electrical performance of the *ϕ*=0.65 composite at stretch ratios of up to *λ*=4 is accompanied by practically unchanged superhydrophobic behaviour through 50 stretch cycles at *λ*=6 (Fig. 4, bottom), further emphasizing the value of the present elastomeric composite for technological applications where simultaneously low electrical resistance and liquid repellency are required.

## Discussion

The composites comprised of CNFs and PF, attaining both superhydrophobic and elastomeric properties, demonstrate high performance and recoverability under severe conditions. Materials of this type have great potential in flexible fabric electronics, where durable resistance to deformation is an intrinsic requirement. Future modifications to such composites will incorporate doping and/or catalyst nanoparticles for added functionality. Another possible application of these conductive composites is in electromagnetic interference shielding^56^. It is finally noted that the present method of solution-processing PF, even within a multi-polymer blend, could be useful in other applications requiring a highly elastomeric matrix.

In summary, we present novel, electrically conductive, adherent composites that are capable of sustaining severe elastic deformation (strains up to 500%) with uncompromised superhydrophobicity and recoverable electrical performance (sheet resistance values of the order of 10^1^–10^2^ Ω sq^−1^). The composites consist of common inexpensive materials applied in a single step as a paint. The polymer matrix, formed from PF, is produced commercially in large quantities and ubiquitous in laboratory environments worldwide. The carbon nanofibre filler is also mass produced and creates excellent charge-conducting percolating pathways, even under extreme strain, with the added role of imparting re-entrant nanoscale surface roughness to the composite (as required for robust superhydrophobicity). Not only are the composites demonstrably functional under severe strains, but also substrate-independent and scalable for high-throughput processing, making them attractive for light-weight, flexible and all-weather electronics. According to current trends favouring flexible devices, a durable and extremely elastomeric composite material with high conductivities that is also water repellent could find use in adaptable, corrosion-resistant electronics or other applications where flexible electrodes are required.

## Methods

### Colloidal solution preparation

Colloidal CNF–PF preparation with 5 wt.% total solids in toluene: a 10 wt.% stock solution of PF solids (Cole-Parmer, Parafilm Wrap PM992, 2″ Wide; 250 Ft/Roll) in toluene (Sigma-Aldrich, 244511; anhydrous, 99.8%) was prepared first. The sealed suspension was heated at 75 °C under mechanical mixing for ∼1 h, or until the PF dissolved completely to form a light grey viscous suspension. Composite ratios are referred to by their particle filler (CNFs) mass fraction *ϕ*=*m*_CNF_/*m*_CNF+PF_ (that is, particle filler mass (*m*_CNF_) divided by total solids mass (*m*_CNF+PF_, combined particle filler and polymer mass)). For example, to prepare 20 g of sprayable colloidal dispersion for a 50 wt.% CNF (*ϕ*=0.5) composite, 0.5 g of CNFs (Applied Sciences, Pyrograf III, PR-24-XT-HHT CNFs, 40–100 nm diameter,<30 μm length after sonication) were weighed out in a 20 ml glass vial. A total of 14.5 g of toluene was added and the mixture was probe sonicated (Sonics & Materials, 750 W, 13 mm probe diameter, 20% amplitude, 20 kHz) for 1.5 min (the remainder of the toluene in the final dispersion derives from the 10 wt.% PF stock solution). A total of 5 g of 10 wt.% PF in toluene was added drop-wise to the probe-sonicated suspension under mechanical mixing and subsequently bath-sonicated (Branson 8,200, 20 kHz, 450 W) for 5 min to ensure a homogenous final dispersion. The total solids content of the final dispersion was fixed at 5 wt.% to avoid high viscosities and spray-orifice clogging. The CNF to PF wt. ratio was varied from 0:100 to 80:20, or in terms of *ϕ*, 0 to 0.8. The all-PF (*ϕ*=0) coating served as control.

### Sample preparation

Strips (1.3 × 7.5 cm^2^) of natural rubber (McMaster-Carr, 86085K101; Abrasion-Resistant Natural Latex Rubber Sheet 1/16″ thick, 12″ × 12″) were cut and mounted in a 7.5 × 9-cm^2^ spray area to a cardboard backing for spraying. The as-received rubber strips had both a rough and a smooth side; only the smooth side was coated with the composite film to avoid added texture from the roughness of the underlying substrate. CNF–PF dispersions were sprayed (Blick Art Supplies 25010-0300; VL-3 siphon-feed Airbrush) onto rubber strips from a distance of ∼25 cm to allow adequate spray atomization before impact on the substrate. The sprayed samples were allowed 24 h in a fume hood to dry before testing to ensure adequate removal of residual solvent.

### Stretch tests and sample characterization

Coated rubber strips were clamp-mounted on a motorized slide (Vexta stepping motor, model PX245, see Supplementary Fig. 5) with an initial 2.5 cm gap (*λ*=1). One clamped end was held stationary, while the other was withdrawn slowly in increments of 2.5 cm (corresponding to *λ*=2, 3 and so on) for stretch, and allowed to return incrementally for relaxation. The stretch ratio *λ* can easily be converted to strain by *e*=*λ*−1. Stretch speeds were also varied within the capabilities of the motor (∼0.1 m s^−1^) and found to have a negligible impact on performance; the stretch speed was thus held constant at ∼5 mm s^−1^. At each interval of elongation, or *λ*, four-probe resistance (HP 34401A Multimeter), dynamic CA and roll-off angle measurements were performed. To attain composite sheet resistance (*R*_s_, units of Ω per square or Ω sq^−1^), two thin strips (∼0.25-cm wide) of silver paint (Ted Pella, Prod. # 16034, 15 g Pelco Colloidal Silver) were applied across the width of the rubber strips orthogonal to the direction of stretch serving as contact points for the multimeter leads, forming a uniform square of conductive composite in between. The thickness and width of the substrates were observed to decline as length increased during elongational stretching. Thus, to ensure accurate *R*_s_ measurements, the distance in the direction of stretch between the two strips of silver paint used for four-probe contact points was equivalent to the corresponding substrate width at various elongations. Five identical samples for each composite ratio were characterized. Three separate sheet resistance measurements were averaged at each *λ*-value for all *ϕ*.

Deionized water was used as the probe liquid for dynamic water droplet CA and roll-off angle measurements. Advancing and receding CA measurements were performed *in situ* on the motorized slide at all *λ*-intervals; water droplets were syringe dispensed onto the composite surface, and similarly withdrawn, using a ∼1 mm *A* (18 gauge) needle tip, such that probe droplet diameters ranged from 1 to 4 mm. For droplet roll-off angle measurements, the motorized slide was mounted on a tilt stage with ∼10 μl droplets placed on the level composites. The slide was tilted until the droplets were observed to roll off, thus designating roll-off angle (*α*) for each *λ*-value.

SEM (Hitachi S3000N VPSEM) was used to image the morphology of the composites from an unstretched state (*λ*=1), intermediate to final strain ratios (*λ*=2,…,6), and after relaxing to an unstretched state from max deformation (*λ*=6→1). Special SEM stubs were machined in-house for imaging under different stretch ratios (Supplementary Fig. 6) after sample removal from the motorized stretch apparatus. Similar specialized SEM stubs were designed to facilitate cross-sectional profile images (Supplementary Fig. 7). Side-view composite thickness estimates were used to determine the extent of elongational composite thinning during strain cycles. Estimates of composite thickness, *t*, allowed for conductivity (*σ*, in S m^−1^) calculations when coupled with sheet resistance *R*_s_, as *σ*=*ρ*^−1^=(*R*_s_ *t*)^−1^; where *σ* and *ρ* are conductivity and resistivity, respectively.

DSC was carried out in a Perkin Elmer DSC7 in flowing nitrogen (20 ml min^−1^). Each sample was heated from 25 to 160 °C at a heating rate of 10 °C min^−1^ and then cooled at the same rate. Thermal properties, such as melting temperature and enthalpy of melting, were determined from a subsequent heating run. For each composition, three samples were analysed.

## Additional information

**How to cite this article:** Mates, J. E. *et al*. Extremely stretchable and conductive water-repellent coatings for low-cost ultra-flexible electronics. *Nat. Commun.* 6:8874 doi: 10.1038/ncomms9874 (2015).

## Supplementary Material

Supplementary Information Supplementary Figures 1-7

## References

[b1] Dalmas F., Cavaille J.-Y., Gauthier C., Chazeau L. & Dendievel R. Viscoelastic behavior and electrical properties of flexible nanofiber filled polymer nanocomposites. Influence of processing conditions. Compos. Sci. Technol. 67, 829–839 (2007).

[b2] Shin M. K. . Elastomeric conductive composites based on carbon nanotube forests. Adv. Mater. 22, 2663–2667 (2010). 20446303 10.1002/adma.200904270

[b3] Chun K.-Y. . Highly conductive, printable and stretchable composite films of carbon nanotubes and silver. Nat. Nanotechnol. 5, 853–857 (2010). 21113161 10.1038/nnano.2010.232

[b4] Brook I., Mechrez G., Suckeveriene R. Y., Tchoudakov R. & Narkis M. A novel approach for preparation of conductive hybrid elastomeric nano-composites. Polym. Adv. Technol. 24, 758–763 (2013).

[b5] Kim K.-S., Jung K.-H. & Jung S.-B. Design and fabrication of screen-printed silver circuits for stretchable electronics. Microelectron. Eng. 120, 216–220 (2014).

[b6] Woo S.-J., Kong J.-H., Kim D.-G. & Kim J.-M. A thin all-elastomeric capacitive pressure sensor array based on micro-contact printed elastic conductors. J. Mater. Chem. C 2, 4415–4422 (2014).

[b7] Gaikwad A. M. . Highly stretchable alkaline batteries based on an embedded conductive fabric. Adv. Mater. 24, 5071–5076 (2012). 22760812 10.1002/adma.201201329

[b8] Yan C. . Stretchable silver-zinc batteries based on embedded nanowire elastic conductors. Adv. Energy Mater. 4, 1301396 (2014).

[b9] Xu S. . Stretchable batteries with self-similar serpentine interconnects and integrated wireless recharging systems. Nat. Commun. 4, 1543 (2013). 23443571 10.1038/ncomms2553

[b10] Li M. . Electrochemical deposition of conductive superhydrophobic zinc oxide thin films. J. Phys. Chem. B 107, 9954–9957 (2003).

[b11] Zhu Y. . Stable, superhydrophobic, and conductive polyaniline/polystyrene films for corrosive enviromnents. Adv. Funct. Mater. 16, 568–574 (2006).

[b12] Das A., Megaridis C. M., Liu L., Wang T. & Biswas A. Design and synthesis of superhydrophobic carbon nanofiber composite coatings for terahertz frequency shielding and attenuation. Appl. Phys. Lett. 98, 174101 (2011).

[b13] Schutzius T. M., Tiwari M. K., Bayer I. S. & Megaridis C. M. High strain sustaining, nitrile rubber based, large-area, superhydrophobic, nanostructured composite coatings. Composites Part A 42, 979–985 (2011).

[b14] Vosgueritchian M., Lipomi D. J. & Bao Z. Highly conductive and transparent PEDOT:PSS films with a fluorosurfactant for stretchable and flexible transparent electrodes. Adv. Funct. Mater. 22, 421–428 (2012).

[b15] Lee P. . Highly stretchable and highly conductive metal electrode by very long metal nanowire percolation network. Adv. Mater. 24, 3326–3332 (2012). 22610599 10.1002/adma.201200359

[b16] Zang J. . Multifunctionality and control of the crumpling and unfolding of large-area graphene. Nat. Mater. 12, 321–325 (2013). 23334002 10.1038/nmat3542 PMC3605241

[b17] Woo J. Y., Kim K. K., Lee J., Kim J. T. & Han C.-S. Highly conductive and stretchable Ag nanowire/carbon nanotube hybrid conductors. Nanotechnology 25, 285203 (2014). 24971604 10.1088/0957-4484/25/28/285203

[b18] Yao S. & Zhu Y. Nanomaterial-enabled stretchable conductors: strategies, materials and devices. Adv. Mater. 27, 1480–1511 (2015). 25619358 10.1002/adma.201404446

[b19] Guo C. F. & Ren Z. Flexible transparent conductors based on metal nanowire networks. Mater. Today 18, 143–154 (2015).

[b20] Cho S. J., Nam H., Ryu H. & Lim G. A rubberlike stretchable fibrous membrane with anti-wettability and gas breathability. Adv. Funct. Mater. 23, 5577–5584 (2013).

[b21] Yang Y., Ruan G., Xiang C., Wang G. & Tour J. M. Flexible three-dimensional nanoporous metal-based energy devices. J. Am. Chem. Soc. 136, 6187–6190 (2014). 24735477 10.1021/ja501247f

[b22] Grobert N. Carbon nanotubes - becoming clean. Mater. Today 10, 28–35 (2007).

[b23] Al-Saleh M. H. & Sundararaj U. A review of vapor grown carbon nanofiber/polymer conductive composites. Carbon 47, 2–22 (2009).

[b24] Kim C. & Zhang S. Mechanical and bending properties of a conductive thin single layer composite consisting of carbon nanofibers and polypyrrole. J Mech. Sci. Technol. 28, 2581–2585 (2014).

[b25] Molnar K. . Enhanced conductivity composites for aircraft applications: carbon nanotube inclusion both in epoxy matrix and in carbonized electrospun nanofibers. Polym. Adv. Technol. 25, 981–988 (2014).

[b26] Elgafy A. & Lafdi K. Effect of carbon nanofiber additives on thermal behavior of phase change materials. Carbon 43, 3067–3074 (2005).

[b27] Cipriano B. H. . Conductivity enhancement of carbon nanotube and nanofiber-based polymer nanocomposites by melt annealing. Polymer 49, 4846–4851 (2008).

[b28] Khattab A., Liu C., Chirdon W. & Hebert C. Mechanical and thermal characterization of carbon nanofiber reinforced low-density polyethylene composites. J. Thermoplast. Compos. Mater. 26, 954–967 (2013).

[b29] Kim H. M. . Electrical conductivity and electromagnetic interference shielding of multiwalled carbon nanotube composites containing Fe catalyst. Appl. Phys. Lett. 84, 589–591 (2004).

[b30] Das A. . Superhydrophobic and conductive carbon nanofiber/PTFE composite coatings for EMI shielding. J. Colloid Interface Sci. 353, 311–315 (2011). 20889160 10.1016/j.jcis.2010.09.017

[b31] Hsieh C. T. & Fan W. S. Superhydrophobic behavior of fluorinated carbon nanofiber arrays. Appl. Phys. Lett. 88, 243120 (2006).

[b32] Song H. J., Shen X. Q. & Meng X. F. Superhydrophobic surfaces produced by carbon nanotube modified polystyrene composite coating. J. Dispersion Sci. Technol. 31, 1465–1468 (2010).

[b33] Yang Y. L., Gupta M. C., Dudley K. L. & Lawrence R. W. Conductive carbon nanofiber-polymer foam structures. Adv. Mater. 17, 1999–2003 (2005).

[b34] McCullen S. D. . Morphological, electrical, and mechanical characterization of electrospun nanofiber mats containing multiwalled carbon nanotubes. Macromolecules 40, 997–1003 (2007).

[b35] Zhu J. . In situ stabilized carbon nanofiber (CNF) reinforced epoxy nanocomposites. J. Mater. Chem. 20, 4937–4948 (2010).

[b36] Tuteja A., Choi W., Mabry J. M., McKinley G. H. & Cohen R. E. Robust omniphobic surfaces. Proc. Natl Acad. Sci. USA 105, 18200–18205 (2008). 19001270 10.1073/pnas.0804872105 PMC2587612

[b37] Sanusi O., Warzoha R. & Fleischer A. S. Energy storage and solidification of paraffin phase change material embedded with graphite nanofibers. Int. J. Heat Mass Transfer 54, 4429–4436 (2011).

[b38] Zhang K., Han B. & Yu X. Electrically conductive carbon nanofiber/paraffin wax composites for electric thermal storage. Energy Convers. Manage. 64, 62–67 (2012).

[b39] Beineke W. F. Parafilm: new way to wrap grafts. HortScience 13, 284–284 (1978).

[b40] Ewens M. & Felker P. The potential of mini-grafting for large-scale production of Prosopis alba clones. J. Arid Environ. 55, 379–387 (2003).

[b41] Gaskin P., Macmilla J., Firn R. D. & Pryce R. J. Parafilm: convenient source of N-alkane standards for determination of gas chromatographic retention indices. Phytochemistry 10, 1155–1157 (1971).

[b42] Higham S. M. & Edgar W. M. Effects of parafilm and cheese chewing on human dental plaque pH and metabolism. Caries Res. 23, 42–48 (1989). 2920383 10.1159/000261153

[b43] Salamzadeh J., Dadashzadeh S., Habibi M. & Estifaie S. Serum and saliva theophylline levels in adult outpatients with asthma and chronic obstructive pulmonary disease (COPD): a cross-sectional study. Iran. J. Pharm. Res. 7, 83–87 (2008).

[b44] Javaherian S., O'Donnell K. A. & McGuigan A. P. A fast and accessible methodology for micro-patterning cells on standard culture substrates using parafilm (TM) inserts. PLoS ONE 6, e20909 (2011). 21687691 10.1371/journal.pone.0020909 PMC3110254

[b45] Lau C., Butenhoff J. L. & Rogers J. M. The developmental toxicity of perfluoroalkyl acids and their derivatives. Toxicol. Appl. Pharmacol. 198, 231–241 (2004). 15236955 10.1016/j.taap.2003.11.031

[b46] Schutzius T. M., Bayer I. S., Qin J., Waldroup D. & Megaridis C. M. Water-based, nonfluorinated dispersions for environmentally benign, large-area, superhydrophobic coatings. ACS Appl. Mater. Interfaces 5, 13419–13425 (2013). 24295138 10.1021/am4043307

[b47] Steele A., Bayer I. & Loth E. Inherently superoleophobic nanocomposite coatings by spray atomization. Nano Lett. 9, 501–505 (2009). 19099463 10.1021/nl8037272

[b48] Wu W. C., Wang X. L., Liu X. J. & Zhou F. Spray-coated fluorine-free superhydrophobic coatings with easy repairability and applicability. ACS Appl. Mater. Interfaces 1, 1656–1661 (2009). 20355780 10.1021/am900136k

[b49] Schutzius T. M., Bayer I. S., Tiwari M. K. & Megaridis C. M. Novel fluoropolymer blends for the fabrication of sprayable multifunctional superhydrophobic nanostructured composites. Ind. Eng. Chem. Res. 50, 11117–11123 (2011).

[b50] Mates J. E. . Water-based superhydrophobic coatings for nonwoven and cellulosic substrates. Ind. Eng. Chem. Res. 53, 222–227 (2014).

[b51] Mates J. E., Schutzius T. M., Qin J., Waldroup D. E. & Megaridis C. M. The fluid diode: tunable unidirectional flow through porous substrates. ACS Appl. Mater. Interfaces 6, 12837–12843 (2014). 24988368 10.1021/am5028204

[b52] Koerner H., Price G., Pearce N. A., Alexander M. & Vaia R. A. Remotely actuated polymer nanocomposites—stress-recovery of carbon-nanotube-filled thermoplastic elastomers. Nat. Mater. 3, 115–120 (2004). 14743213 10.1038/nmat1059

[b53] Lipomi D. J. . Skin-like pressure and strain sensors based on transparent elastic films of carbon nanotubes. Nat. Nanotechnol. 6, 788–792 (2011). 22020121 10.1038/nnano.2011.184

[b54] Mhike W., Focke W. W., Mofokeng J. P. & Luyt A. S. Thermally conductive phase-change materials for energy storage based on low-density polyethylene, soft Fischer–Tropsch wax and graphite. Thermochim. Acta 527, 75–82 (2012).

[b55] Cassie A. B. D. & Baxter S. Wettability of porous substrates. Trans. Faraday Soc. 40, 546–551 (1944).

[b56] Liu L., Das A. & Megaridis C. M. Terahertz shielding of carbon nanomaterials and their composites: a review and applications. Carbon 69, 1–16 (2014).

