# Supplementary material for: Extremely stretchable and conductive water-repellent coatings for low-cost ultra-flexible electronics
Source: Nat Commun. 2015 Nov 23;6:8874. doi: 10.1038/ncomms9874 (PMC4673499; doi:10.1038/ncomms9874)
Supplement: Supplementary Information — Supplementary Figures 1-7 [file ncomms9874-s1.pdf]

Supporting Information

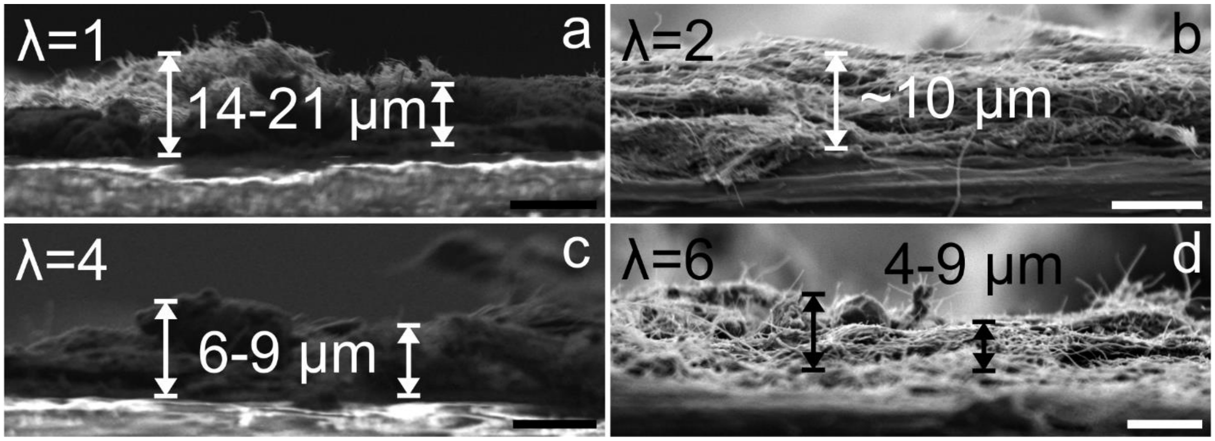

Supplementary Figure 1 | Profile SEM images. Typical cross-sectional profile SEM images of a stretch sequence for the $\varphi = 0.5$ composite applied on natural rubber substrate. Images were used to approximate composite thickness under strain (*i.e.*, elongational thinning): (**a**) $\lambda = 1$, average coating thickness ~20 µm (20 µm scalebar); (**b**) $\lambda = 2$, average coating thickness ~10 µm (10 µm scalebar); (**c**) $\lambda = 4$, average coating thickness ~7 µm (10 µm scalebar); (**d**) $\lambda = 6$, average coating thickness ~5 µm (10 µm scalebar). To convert sheet resistance to conductivity, a more accurate volumetric representation of composite performance, the approximate composite thicknesses were calculated using the power law curve fitted in Supplemental Figure 2 ($t_{stretch} = 24.2\ \lambda^{-1.2}$). Assuming conservation of mass, this power law curve fit was also used to estimate composite thickness during relaxation from stretched states. The curve-fitted thickness values were coupled with the measured R_s values to derive composite conductivities (σ), as shown in Figure 2c.

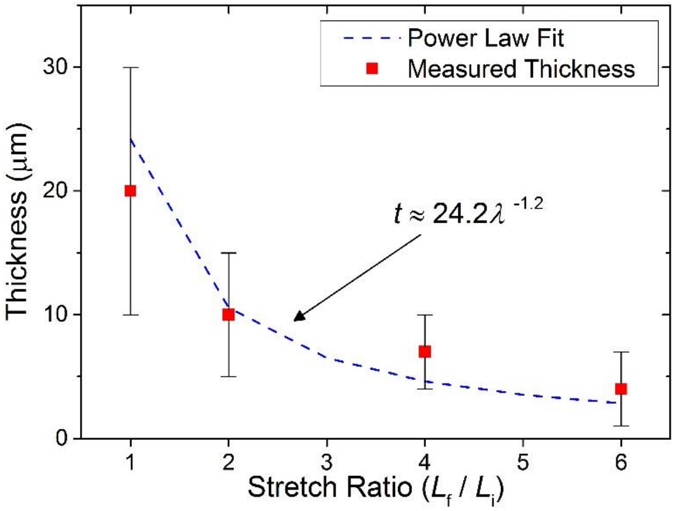

Supplementary Figure 2 | Coating thickness *vs.* sample elongation. Measured average thickness (t) values of the composite (red squares) at different stretch ratios λ. Elongational thinning follows a power law curve, shown by the blue dashed line. The power law equation fitted to the experimental data is used to estimate thickness for composite coating volume. Assuming mass conservation, this curve fit was also used for the relaxation cycles ($\lambda = 6 \rightarrow 1$).

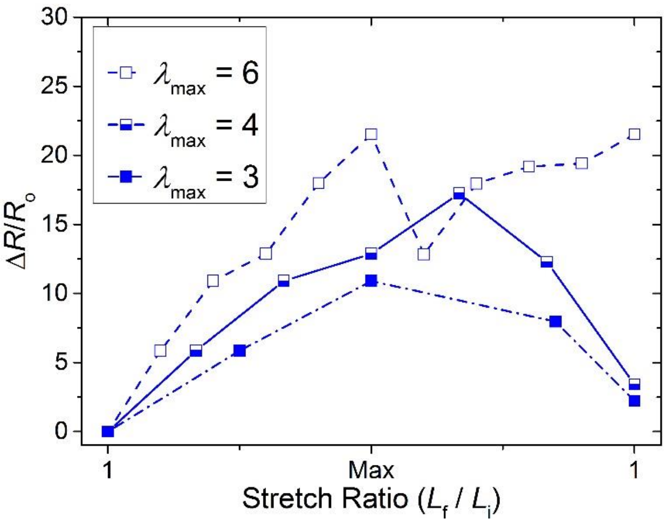

Supplementary Figure 3 | Relative electrical performance. Sheet resistance changes relative to initial resistance ($\Delta R/R_o$) for $\varphi = 0.65$, the best performing composite. The corresponding absolute values are plotted in Figure 2.

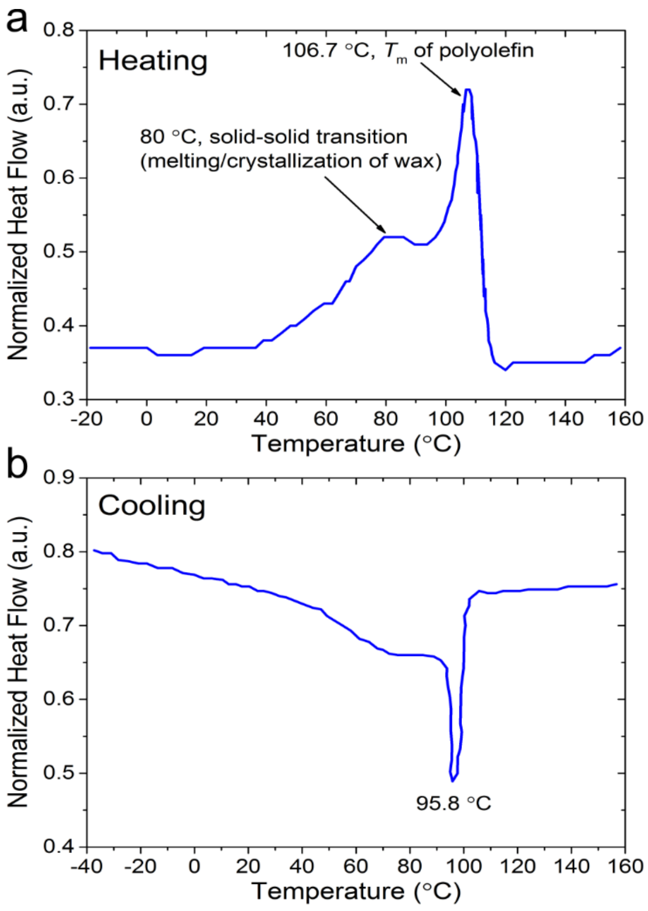

Supplementary Figure 4 | DSC characterization. Thermal properties of PF. (**a**) Two peaks are observed at 80 and 106.7 ºC on the heating curve, representing the solid-solid transition of paraffin wax and the T_m of polyolefin, respectively. Melting enthalpy of the blend was 136 J/g. Crystallization temperatures are 71.6 and 96.1 ºC for wax and polyolefin, respectively. (**b**) The cooling curve of PF. A sharp polyolefin crystallization peak is observed at 95.8 ºC instead. This may be attributed to co-crystallization of higher molecular weight wax in PF with the polyolefin. The main wax component crystallization appears as a broad shoulder between 50 and 75 ºC. This indicates good miscibility between the wax crystals and the polyolefinic component of PF.

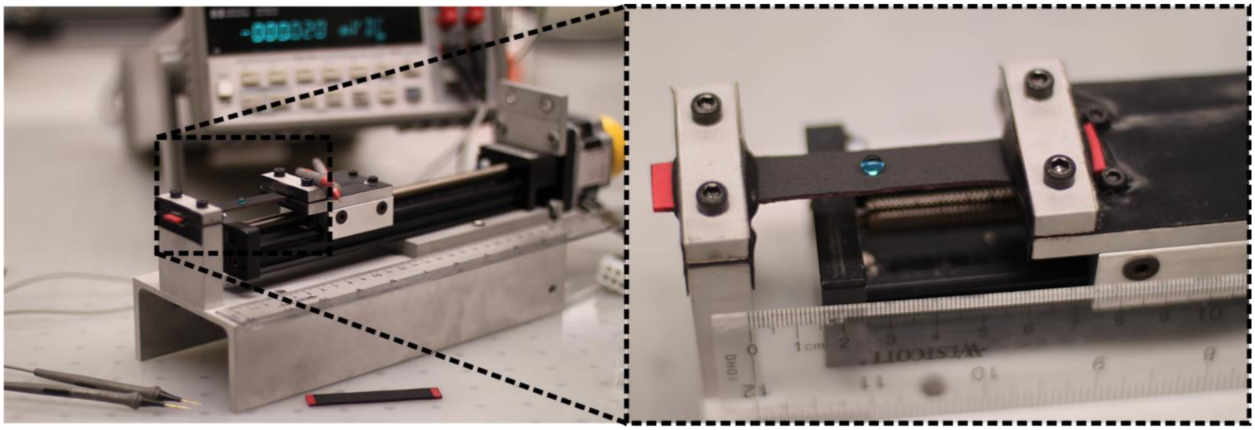

Supplementary Figure 5 | Stretch apparatus with electronic test equipment and sessile beaded droplet. Photographs of the custom-made motorized stretch apparatus used for deforming coated rubber substrates through fixed stretch ratios (λ). The detail on the right shows a top-down view of the stretched composite with a beaded water droplet (dyed blue for visualization) in the center. The metal clamps are padded with rubber to prevent electrical conduction through the apparatus.

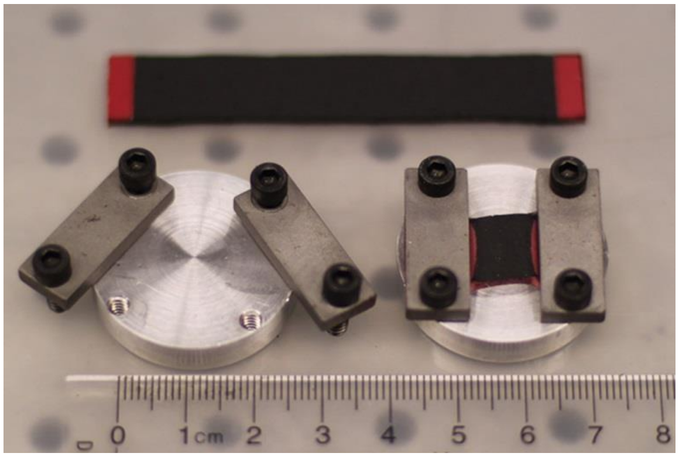

Supplementary Figure 6 | Custom-made sample mounting stages. The SEM stubs maintain the sample's stretched state after removal from the motorized slide, thus allowing SEM imaging of composite deformation. On top of the image (out of focus strip) is an example of a coated rubber substrate. The stub on the left is empty to display how the clamps swivel for substrate attachment. The stub on the right shows a coated sample secured at stretch ratio $\lambda = 2$.

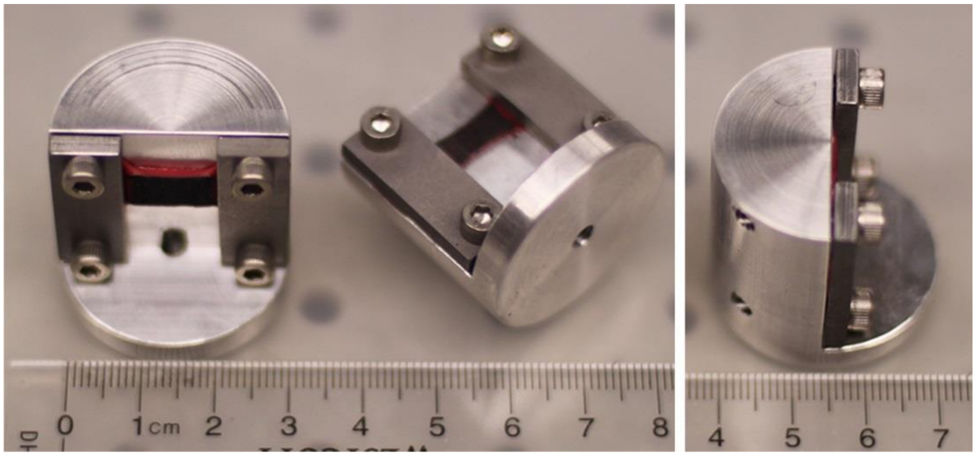

Supplementary Figure 7 | Custom-made profile SEM mounting stages. Similar SEM stubs as those shown in Supplementary Figure 6 were constructed to determine thickness profiles of the composites through various stretch ratios. The coating thickness values were used to quantify volumetric conductivity values (Figure 2 and 4).
